# Tolerable pain reduces gastric fundal accommodation and gastric motility in healthy subjects: a crossover ultrasonographic study

**DOI:** 10.1186/s13030-017-0089-5

**Published:** 2017-02-01

**Authors:** Hideaki Hasuo, Hiroaki Kusunoki, Kenji Kanbara, Tetsuya Abe, Naoko Yunoki, Ken Haruma, Mikihiko Fukunaga

**Affiliations:** 1 Department of Psychosomatic Medicine, Kansai Medical University, Shinmachi 2-5-1, Hirakata, Osaka Japan; 2 Department of General Medicine, Kawasaki Medical Univesrity, Matsushima 577, Kurashiki, Okayama Japan; 3 Department of Internal Medicine, Akaiwa Medical Association Hospital, Shimoichi 187-1, Akaiwa, Okayama Japan

**Keywords:** Tolerable pain, Gastric fundal accommodation, Gastric motility, Pain management, Ultrasonography

## Abstract

**Background:**

Obstacles to pain management include patients’ reluctance to inform healthcare provides about their pain, and differences in the pain management aims between patients and healthcare providers. The objective of this study was to evaluate whether tolerable pain influences gastric fundal accommodation and gastric motility in healthy subjects.

**Methods:**

We undertook a crossover comparison study to evaluate gastric fundal accommodation and gastric motility in 74 healthy subjects in the presence or absence of tolerable pain. The intensity of tolerable pain was defined as the upper limit of pain compatible with comfortable daily life. Pain was generated by clipping a clothes pin to the ear lobe, and the intensity of pain was adjusted by inserting the gauze between the ear lobe and the pin. Gastric fundal accommodation and gastric motility were assessed by external ultrasonography. The cross-sectional area of the proximal stomach was measured after subjects had taken 100 mL-liquid meals four times, then the amplitude and frequency of antral contractions were measured.

**Results:**

The median numerical rating scale of tolerable pain was 3 (interquartile rang 2–4). Gastric fundal accommodation, gastric motility and gastric emptying were all significantly impaired by tolerable pain (*P* < 0.001 for all comparisons).

**Conclusions:**

Even tolerable pain can reduce gastric fundal accommodation and gastric motility, which could result in anorexia or decreased quality of life. Our findings provide important insights into pain management education for patients tolerating pain and healthcare providers encouraging patients to tolerate pain. This study was registered retrospectively.

## Background

Early initiation of appropriate pain management is important, as pain causes stress. Chronic pain has adverse influences on central nociceptive pathways, the descending pain modulatory system and the autonomic nervous system, as well as various psychosocial factors that include anxiety and depression [1, 2].

Patients have reported obstacles to appropriate pain management [3, 4]; many studies have evaluated patients’ concerns about opioid usage for cancer pain relief. Specific questionnaires have been established to measure patients’ perceptions of barriers to care. One of the best known is the Barriers Questionnaire (BQ), which consists of seven themes: fatalism; addiction; side effects; distraction; progression; tolerance, and injection [5]. A cross-sectional study using the BQ reported beliefs that ‘good’ patients do not complain about pain [6]. Consequently, one of the obstacles to appropriate pain management is patient’s reluctance to complain about their pain to healthcare providers. When patients’ reluctance overwhelms disadvantages from tolerating pain, they do not proactively tell healthcare providers about their pain. It can be argued that the intensity of pain that does not raise complains to healthcare providers equates to “tolerable” pain.

To overcome obstacles to appropriate pain management, early initiation of pain management education is important. Better understanding of the disadvantages of tolerating pain encourages patients to tell healthcare providers about it. We hypothesized that demonstrating the negative effects of tolerable pain on mind and body would provide important insights for patients learning about pain management. Anorexia affects 30 to 92% of patients with cancer [7], and many seek relief of anorexia as well as pain. We examined whether providing objective evidence of the adverse influence of tolerable pain on gastrointestinal function would encourage patients hesitating to complain about tolerable pain to take a more proactive approach to pain control.

To the best of our knowledge, the influence of tolerable pain on gastrointestinal function has not been examined. There is, however, evidence from animal models that tolerable pain nonetheless has physiologic consequences, as rat dorsal horn neurons can be sensitized by repeated subthreshold synapse stimulation [8]. A body of evidence has illuminated the relationship between pain and autonomic function [9, 10], and gastrointestinal function [11]. It is well recognized that gastric fundal accommodation and gastrointestinal motility are under the control of the autonomic nervous system [12]. Gastric fundal accommodation is the capacity of stomach to relax, so as to store ingested food, and interaction with the gastric antrum has been also reported [13, 14]. We have established techniques to assess gastric fundal accommodation and gastric motility using external ultrasonography (US) in clinical practice [15–17].

In this study, we objectively assessed the effects of pain that healthy subjects deemed tolerable on gastric fundal accommodation and gastric motility as primary endpoints, measured using external US. In addition, we determined the intensity of pain that the healthy subjects subjectively deemed tolerable on an 11-point numerical rating scale (NRS) as a secondary endpoint.

## Methods

### Participants

We enrolled 74 healthy volunteers to our study: 37 females and 39 males. The median age was 39.5 years (range 20–63 years). None of the subjects had chronic pain or diabetes mellitus. None had undergone gastrointestinal surgery, or was taking any drug that could affect gastric motility.

### Study design

#### Subjective pain assessment

The intensity of pain that study subjects deemed tolerable was determined by subjective pain assessment on the NRS. The intensity of pain that study subjects deemed tolerable was defined as the pain that was nonetheless compatible with a comfortable daily life (i.e. its intensity allowed study subjects to achieve comfort in physical, functional, and psychosocial domains) [18].

#### Objective pain assessment

A crossover comparison study was undertaken to evaluate gastric fundal accommodation and gastric motility with or without tolerable pain. Volunteers underwent ultrasound examination with pain followed by examination without pain, or *vice versa*, alternately based on the order of study enrollment. The interval between examinations with or without tolerable pain was 1–7 days.

**Fig. 1:**
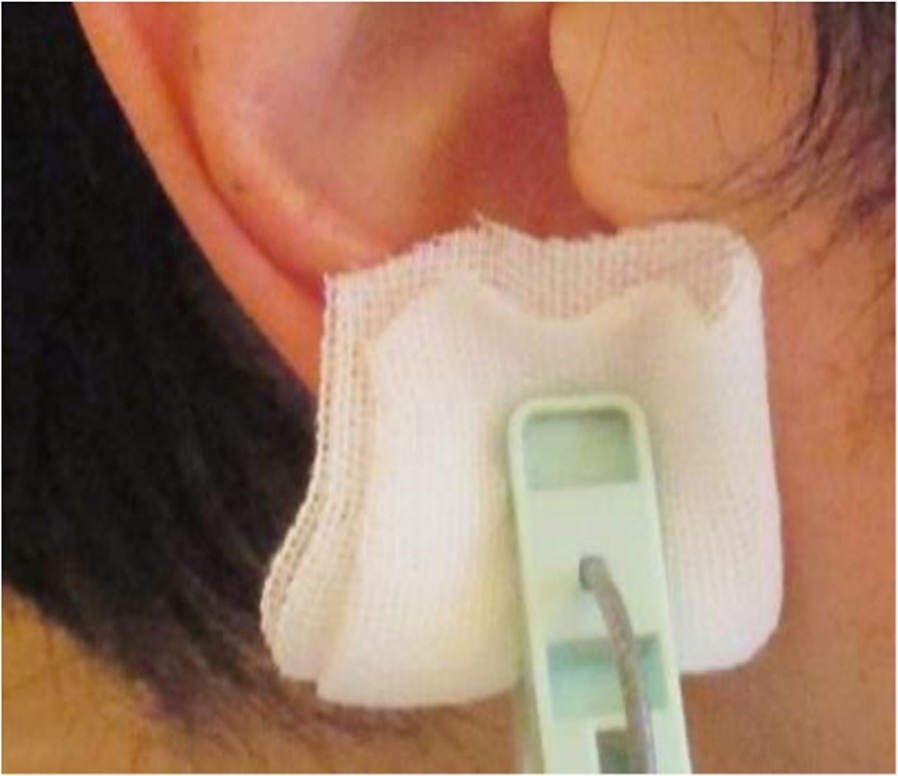
Clothes pin and gauze. Tolerable pain was generated by clipping a clothes pin to an ear lobe. The intensity of pain was adjusted by inserting gauze between the ear lobe and pin

Tolerable pain was generated by clipping a clothes pin (product name: clothing clip, materials: hard plastic, each size: 5.5 × 1.2 × 2.7 cm, weight: 72 g) to an ear lobe, with reference to the definition above. The intensity of pain was adjusted by inserting gauze between the ear lobe and pin (Fig. 1). The maximum of the pressure was assumed when the entire grasping portion of the pin was sandwiched directly between the ear lobe. Once a steady intensity of tolerable pain had been achieved, gastric fundal accommodation and gastric motility were assessed using external US (TUS–A300 US system, Toshiba, Nasu, Japan; 3–MHz curved-array probe). All examinations were carried out by the same researcher (H.H.) to avoid variation in the examination procedure.

### Assessment of gastric fundal accommodation (Fig. 2)

**Fig. 2:**
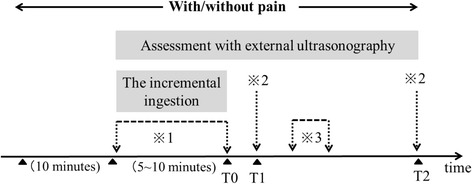
The assessment of gastric fundal accommodation and gastric motility with and without pain. T0: the end of the incremental ingestion. T1: 1–minute after the after the subject had changed position (T0). T2: 15–minutes after the subject had changed position (T0). ※1: measure of the cross–sectional area of the proximal stomach. ※2: measure of the antral cross–sectional area. ※3: measure of the number of contractions and the contraction rate for 3–minute period

The gastric fundal accommodation reflex was assessed by measuring the cross-sectional area of the proximal stomach after incremental ingestion, using a straw, of up to 400 mL of a liquid meal (consommé soup; 13.1 kcal; 400 mL at 37 °C as previously described [17]) with the subject supine on a bed. To obtain the maximal cross-sectional area of the proximal stomach, the US probe was placed and maintained in the intercostal space of the left axilla using the spleen as a landmark. The cross-sectional area of the proximal stomach was quantified by tracing its mucosal side with a built-in caliper for 1–2 min after incremental meal ingestion.

### Assessment of gastric motility (Fig. 2)

After assessment of the gastric fundal accommodation reflex, the subject sat on a chair reclining slightly backwards. Gastric emptying and antral contractions were observed using US as previously described [15, 16]. The US probe was positioned vertically to permit simultaneous visualization of the antrum, the superior mesenteric artery, and the abdominal aorta.

The antral area was estimated by tracing the mucosal side of the antrum with a built-in caliper 1 min and 15 min after the subject had changed position. Gastric emptying rate was calculated as a percentage according to the following formula:

$$ \left[\left(\mathrm{antral}\ \mathrm{area}\ 1\  \min\ \mathrm{after}\ \mathrm{meal}\ \mathrm{ingestion}\ \hbox{--}\ \mathrm{antral}\ \mathrm{area}\ 15\  \min\ \mathrm{after}\ \mathrm{meal}\ \mathrm{ingestion}\right)\ /\ \mathrm{antral}\ \mathrm{area}\ 1\  \min\ \mathrm{after}\ \mathrm{meal}\ \mathrm{ingestion}\right] \times 100\ \left(\%\right) $$

The frequency of antral contractions was defined as the number of contractions per 3-min interval. The amplitude of antral contractions was calculated as a percentage from the maximal reduction in antral area for each contraction, thus:

$$ \left[\left(\mathrm{antral}\ \mathrm{area}\ \mathrm{relaxed}\ \hbox{--}\ \mathrm{antral}\ \mathrm{area}\ \mathrm{contracted}\right)\ /\ \mathrm{antral}\ \mathrm{area}\ \mathrm{relaxed}\right] \times 100\ \left(\%\right) $$

The motility index was the product of the mean amplitude and the frequency of contractions.

### Statistical analyses

Differences were tested using the Wilcoxon signed-rank sum test. Statistical analysis was performed using SigmaStat3.5, and the level of statistical significance was set at *P* < 0.05.

## Results

### Subjective pain assessment

The median intensity of the pain that the subjects deemed tolerable was 3 on the NRS (interquartile range 2–4). Both females and males had similar results. The tolerable NRS score was 1 in 14 subjects (18.9%), 2 in 14 (18.9%), 3 in 27 (36.5%), 4 in seven (9.5%), 5 in eight (10.8%), 6 in three (4.0%), and 7 in one (1.4%).

### Objective assessment of pain on gastric function

**Fig. 3:**
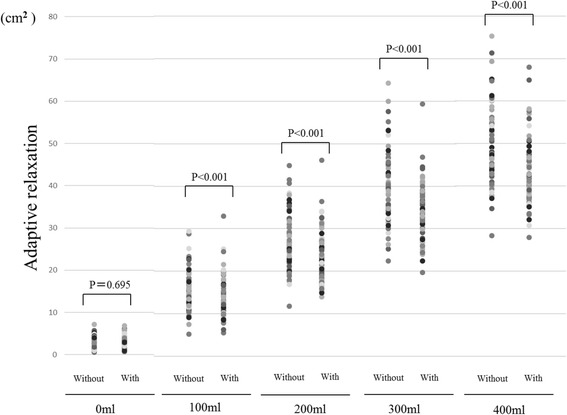
Objective assessment of tolerable pain on gastric fundal accommodation. The maximal cross-sectional areas of the proximal stomach decreased significantly at all measured points when volunteers were subjected to tolerable pain

The influence of tolerable pain on gastric fundal accommodation is shown in Fig. 3. The mean maximal cross-sectional area of the proximal stomach after the ingestion of 0 mL, 100 mL, 200 mL, 300 mL and 400 mL of the liquid meal was 2.5 cm^2^ [standard deviation, SD 1.5] without pain and 2.5 cm^2^ [SD 1. 5] with tolerable pain, 15.2 cm^2^ [SD 4.7] without pain and 13.4 cm^2^ [SD 4.8] with tolerable pain, 27.4 cm^2^ [SD 6.5] without pain and 23.2 cm^2^ [SD 6.0] with tolerable pain, 40.0 cm^2^ [SD 8.0] without pain and 33.6 cm^2^ [SD 6.6] with tolerable pain, and 49.7 cm^2^ [SD 9.1] without pain and 42.8 cm^2^ [SD 7.5] with tolerable pain, respectively. The maximal cross-sectional areas of the proximal stomach decreased significantly at all measured points when volunteers were subjected to tolerable pain.

**Fig. 4:**
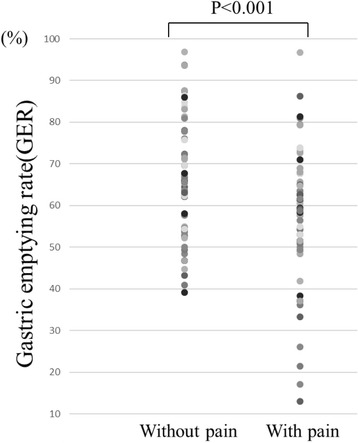
Objective assessment of tolerable pain on gastric emptying rate. Mean gastric emptying rate was significantly higher in those without tolerable pain (65.4% compared with 58.0% in those with pain)

**Fig. 5:**
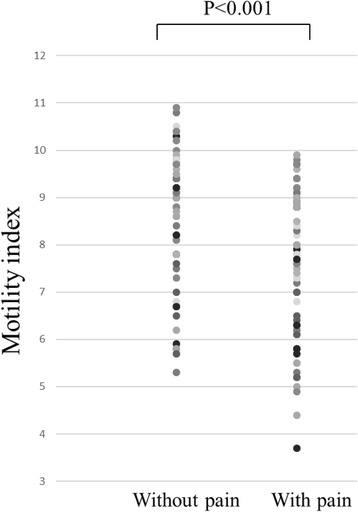
Objective assessment of tolerable pain on motility index. The mean motility index was also significantly higher in those without tolerable pain (8.7 compared with 7.7 in those with pain

Tolerable pain significantly diminished gastric emptying rate and gastric motility index. Mean gastric emptying rate was significantly higher in those without tolerable pain (65.4% [SD 13.2] compared with 58.0% [SD 15.0] in those with pain, *P* < 0.001; Fig. 4). The mean motility index was also significantly higher in those without tolerable pain (8.7 [SD 1.4] compared with 7.7 [SD 1.5] in those with pain, *P* < 0.001; Fig. 5).

There was no significant difference between females and males on the objective assessment of pain on gastric function.

## Discussion

To the best of our knowledge, ours is the first study to have objectively demonstrated that tolerable pain reduces gastric fundal accommodation and gastric motility in healthy volunteers.

We found that the median NRS of pain that healthy subjects deemed tolerable was 3. Although there is no consensus about the precise NRS value of pain tolerability, Serin et al. reported NRS scores of 1–4, 5–6 and 7–10 as mild, moderate and severe pain, respectively [19]. Based on National Comprehensive Cancer Network guidelines, an NRS scores of ≥4 requires consideration of analgesic drugs such as opioids [20]. A median pain intensity rated by healthy subjects of 3 is therefore consistent with tolerability, as it is not sufficiently severe to warrant drug treatment.

Our main aim was to evaluate the influence of pain on gastric fundal accommodation and gastric motility. It has previously been reported that pain reduces autonomic nervous system activity [9, 10]; however, there have been no objective reports illuminating the relationship between pain and gastric fundal accommodation and gastric motility. Our findings suggest that even tolerable pain can affect gastrointestinal function. As the influence of the autonomic nervous system on gastrointestinal function is well known [12], it is likely that the impairment of gastrointestinal function by pain is mediated by the autonomic nervous system.

We elicited nociceptive pain by applying mechanical stimulation to the skin, connective tissue and cartilage of the ear lobe. This stimulation would likely be transmitted to the somatosensory area in the cerebral cortex by Aδ fibers and C fibers of peripheral sensory nerves. C fibers routed through the cerebral limbic system—especially the amygdala (spino-parabrachio-amygdaloid pathways) and thenceforth to the hypothalamus—are thought to play an important part in establishing states of anxiety and distress. Furthermore, there is another direct nociceptive pathway from periphery to hypothalamus (spinohypothalamic pathway). Cerebral activity during pain perception has been called the “Pain Matrix” [21]. We judge that the physiological changes observed in this study were consequences of pain as a stressor acting on the hypothalamus to reduce autonomic nervous system activity and thus gastrointestinal function. Experimentally-induced anxiety has been reported to reduce gastric fundal accommodation in healthy volunteers by means of a physiologic mechanism that include the amygdala, hypothalamus and autonomic nerves system [22]. Fear and desperation caused by pain, which are high-order processing-dependent stressors generated within the cerebral cortex before acting on the amygdala, likely had a negligible effect in this study as the pain was tolerable, of a known cause, and could be terminated on request.

Our second important finding was that even mild pain that healthy subjects deemed tolerable negatively affected gastrointestinal function, which suggests that tolerating pain may cause anorexia and impair quality of life (QOL). Our findings provide important insights that could inform education about pain management strategies for patients tolerating pain. The benefits of educational interventions in patients with cancer have previously been reported [23]; most interventions seek to encourage patients to report the true intensity of their pain. Nonetheless, our findings also underline the importance of not tolerating pain but treating it appropriately. Healthcare providers should be aware that even tolerable pain may impair QOL, removing one of the potential barriers to care that patients encounter. There has been a great deal of recent interest in personalized pain goal to tailor pain management for patients with cancer [18, 24]. The definition of pain used in a study of personalized pain goal used an NRS score of 3 [18], the same as the median pain intensity our volunteers found tolerable. This pain goal, however, was established based on the authors’ assumption that complete pain relief was difficult, we would argue that healthcare providers need to have enthusiasm for providing more aggressive pain relief. It has been reported that pain goals of individual patients are often not clear to the healthcare providers [25, 26]. Green et al. have proposed that identifying a tailored personalized pain goal should be a critical part of pain management education, along with close communication between the patient and healthcare providers [27].

### Limitations

Our study had several limitations. First, the technique of using a clothes pin to stimulate pain has not been validated, and it is more common to induce pain with electric stimulation, which has the additional advantages of being quantifiable and has the ability to differentially stimulate specific peripheral sensory nerve subtypes at different electric frequencies [28]. Our technique was not able to distinguish between peripheral sensory nerve subtypes. Our subjects subjectively judged pain stimulation threshold, however, thereby eliminating the need for quantitative pain stimulation. Furthermore, our technique allowed us to apply a painful stimulus continuously for more than 30 min so as to measure gastric function; this duration of stimulation would not have been possible with an electric pain inducer. Second, we didn’t set a clipping condition without feeling ear pain as sham stimulus. Clipping a pin to an ear per se might distract our subject’s attention to drinking a liquid meal and affect gastric fundal accommodation and gastric motility regardless of ear pain. We also didn’t assess gastrointestinal symptoms and anxiety level before and during the meal ingestion. The reason was that this study was a crossover comparison one and that the assessment during the meal ingestion might be actually difficult. Third, gastrointestinal function is influenced by the endocrine and immune systems as well as the autonomic nervous system, so the changes in gastrointestinal function observed in this study may not solely be explained by the influence of the autonomic nervous system. We adjunctively should have recorded heart rate and blood pressure. It has been already suggested that pain influences heart rate and blood pressure through a somato-autonomic reflex [29]. We did not seek, however, to explore the physiologic mechanism underpinning the changes we found, instead focusing on providing evidence to inform appropriate pain management education for patients tolerating pain. In the future, other autonomic functions such as heart rate variability and salivary amylase concentration could be measured to illuminate further the role of the autonomic nervous system in modulating the effect of pain on the gastrointestinal system. Finally, we were unable to control for observer bias, as our pain stimulus could not be blinded.

## Conclusions

Even tolerable pain can reduce gastric fundal accommodation and gastric motility, which in long term could result in anorexia or decreased QOL. Our findings provide important insights to inform pain management education for patients tolerating pain and healthcare providers encouraging patients to tolerate pain.

## Abbreviations

BQ: Barriers Questionnaire
IQR: Interquartile range
NRS: Numerical rating scale
QOL: Quality of life
US: Ultrasonography
